# Recent insights in nanotechnology-based drugs and formulations designed for effective anti-cancer therapy

**DOI:** 10.1186/s12951-016-0193-x

**Published:** 2016-05-26

**Authors:** Ewelina Piktel, Katarzyna Niemirowicz, Marzena Wątek, Tomasz Wollny, Piotr Deptuła, Robert Bucki

**Affiliations:** Department of Microbiological and Nanobiomedical Engineering, Medical University of Bialystok, Mickiewicza 2c, 15-222 Bialystok, Poland; Holy Cross Oncology Center of Kielce, Artwińskiego 3, 25-317 Kielce, Poland; Department of Physiology, Pathophysiology and Immunology of Infections, The Faculty of Health Sciences of the Jan Kochanowski University in Kielce, Kielce, Al. IX Wieków Kielc 19, 25-317 Kielce, Poland

**Keywords:** Cancer, Nanotechnology, Drug delivery

## Abstract

The rapid development of nanotechnology provides alternative approaches to overcome several limitations of conventional anti-cancer therapy. Drug targeting using functionalized nanoparticles to advance their transport to the dedicated site, became a new standard in novel anti-cancer methods. In effect, the employment of nanoparticles during design of antineoplastic drugs helps to improve pharmacokinetic properties, with subsequent development of high specific, non-toxic and biocompatible anti-cancer agents. However, the physicochemical and biological diversity of nanomaterials and a broad spectrum of unique features influencing their biological action requires continuous research to assess their activity. Among numerous nanosystems designed to eradicate cancer cells, only a limited number of them entered the clinical trials. It is anticipated that progress in development of nanotechnology-based anti-cancer materials will provide modern, individualized anti-cancer therapies assuring decrease in morbidity and mortality from cancer diseases. In this review we discussed the implication of nanomaterials in design of new drugs for effective antineoplastic therapy and describe a variety of mechanisms and challenges for selective tumor targeting. We emphasized the recent advantages in the field of nanotechnology-based strategies to fight cancer and discussed their part in effective anti-cancer therapy and successful drug delivery.

## Background

**Fig. 1:**
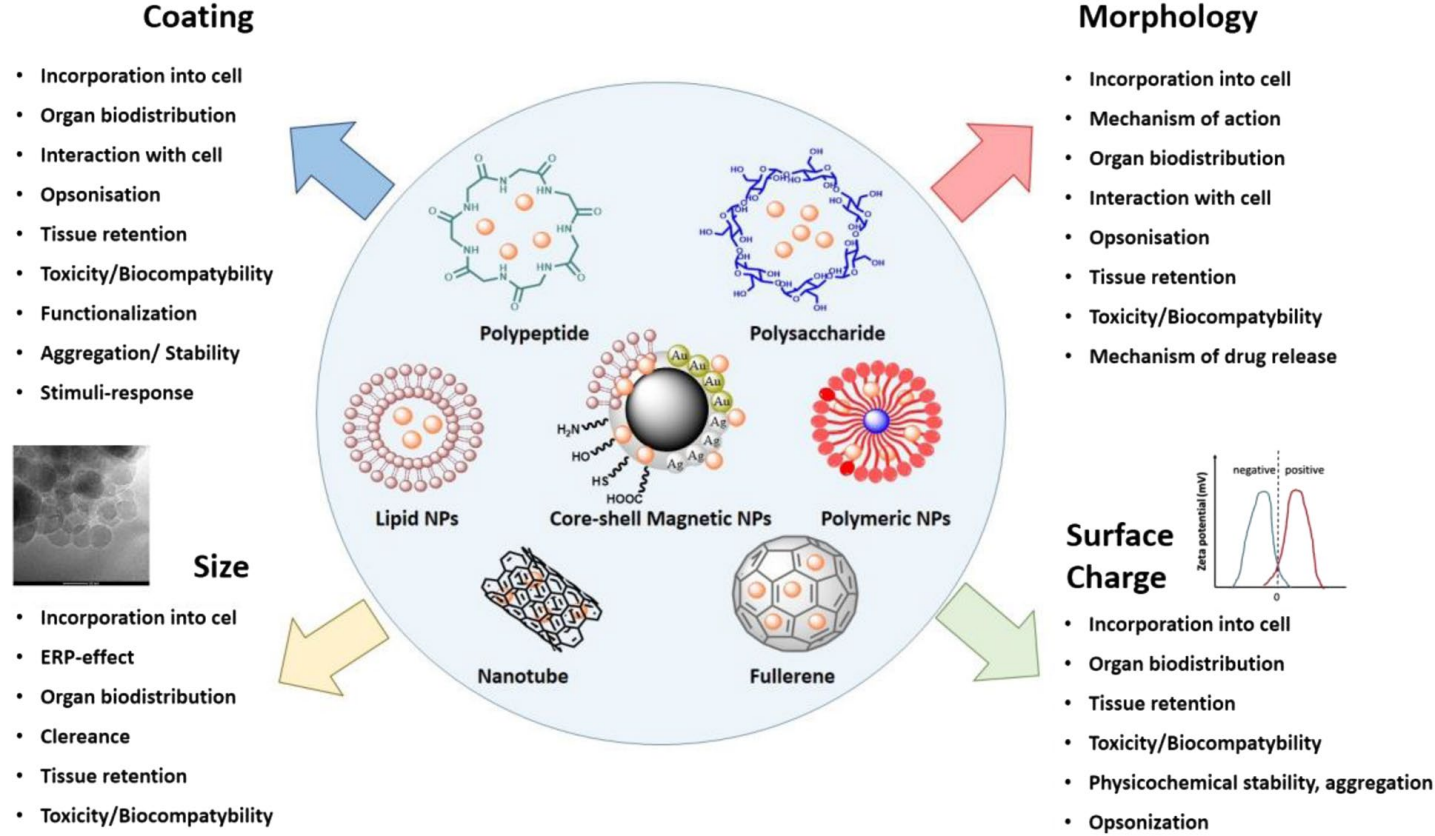
Physicochemical features of different nanomaterials proposed as drug carriers in drug delivery systems and targeted therapy. The most important properties of nanomaterials determining their theranostic potential, employment in medical applications and effect on pharmacokinetic parameters in vivo conditions, including biodistribution, toxicity and internalization into target cells

Despite the continuous improvement of cancer fighting strategies, malignancies are one of the leading causes of death worldwide. Over the last decades a number of novel antineoplastic compounds, acting through induction of apoptosis, dysfunction in cell cycle, gene transcription and inhibition of angiogenesis process, have been presented [1]. Nevertheless, the standard anti-cancer treatment is still based on combined surgical intervention, radiation and chemotherapy. The use of these methods is limited due to anti-cancer drugs toxicity, their poor selectivity, possibility of cancer recurrence and the induction of drug-resistant cancer cells [2]. The growing number of studies confirmed that big part of these limitations might be overcome using new nanotechnology-based tools [2, 3]. A variety of nanostructures including synthetic biodegradable polymers, such as chitosan (CS), polycaprolactone (PCL) or poly-lactic-co-glycolic acid (PLGA), lipids (liposomes, nano-niosomes, solid-lipid nanoparticles), mesoporous silica nanoparticles (MSNs), micelles, quantum dots (QDs), carbon nanotubes (CNTs) and iron oxide magnetic nanoparticles (MNPs) have been investigated [4–10]. It is recognized, that nanoparticles exhibit the medical potential due to a broad spectrum of unique physicochemical and biological features including large surface/volume ratio, specific structural properties, an ability to attach some specific agents on their surface, capability to cross cell or tissues barriers and long circulation time in blood when compared to other particles. The summary of properties determining the employment of nanostructures in medical application is presented in Fig. 1. It is well established that nanoparticles’ small sizes facilitate their administration through oral, nasal, parenteral and intraocular routes [2]. In addition, their internalization via endocytosis, phagocytosis, pinocytosis and macropinocytosis process is possible as well [11]. A number of studies confirmed that size, shape, hydrodynamic diameter and properties of nanoparticles’ surface determine their residual time in blood, their renal clearance, protein absorption, toxicity, uptake into mammalian cells and in vivo tumor targeting efficiency [12–18]. Interestingly, the study by Palanki et al. revealed, that size of silver nanoparticles (AgNPs) determines the chemopreventive effectiveness of AgNPs against UVB-induced DNA damage during apoptosis and influences the protective effect of these structures against skin cancers [19]. It was also demonstrated that surface properties of nanomaterials govern physicochemical stability. High positive or negative zeta potential is associated with their stability preventing accumulation of stored materials. Moreover, recent study conducted by Yang et al. confirmed that positively charged gold nanoparticles (AuNPs) are better internalized by breast cancer cells than particles with negative charge [20]. The character of surface charge represents an important parameter determining biological activity of nanomaterials [21–23]. The size of MNPs’ core influences also magnetic properties of these structures which are crucial for their employment in magnetic fluid hyperthermia (MFH), magnetic resonance imaging (MRI) and magnetic-mediated targeted delivery [24]. Simultaneously, amphipathic properties of lipid-based nanostructures, such as liposomes or micelles, controls the payload drugs accumulation into tumor tissues and are suitable to deliver drugs characterized by low solubility in water environment and agents of various chemical nature [25, 26].

Unique nanomaterials properties makes possible to employ nanostructures in a number of biological applications, such as drug delivery systems (DDS), contrast agents for MRI or computer tomography (CT) imaging and as diagnostic tools for high-specific detection of macromolecules and pathogens [3, 27–29]. In this review, we summarize the potential of nanotechnology in modern therapy of cancer diseases and highlight the recent advantages in this field. In addition, we discussed the studies using nanomaterials suitable for medical application and described a variety of mechanisms allowing their employment as nanostructures to overcome some limitations of conventional anti-cancer methods. Firstly, we present data confirming the possibility of nanotechnology application in order to improve selectivity of antineoplastic compounds via passive and active drug delivery strategy, using monoclonal antibodies, aptamers, nucleic acid, peptides and stimuli-responsive nanocarriers. Next, we discuss the nanotechnology-based approaches to overcome the chemotherapeutics resistance by preventing drugs efflux and enhancing the intracellular uptake, modulating ceramide levels and targeting MDR-associated genes and proteins. Furthermore, we highlighted the recent advantages in design of antineoplastic strategies in order to improve pharmacokinetic parameters of anti-cancer drugs, increasing their low water-solubility, optimization of its controlled release and upgrading of oral bioavailability and chemical stability. We also discussed how physical properties of nanomaterials might contribute to intensification of standard anti-cancer procedures. With respect to considerable achievements in nanotechnology-based strategies, we bring a brief summary of limitations facing nanotechnology-based therapies and governing pre-clinical studies and summarized the results of newest clinical trials after their translation in clinical settings.

## Nanomaterials-based targeted drug delivery systems to increase low selectivity of chemotherapeutics

Therapeutic agents’ delivery to the target site is a major challenge in the treatment of a variety of diseases, including cancer, and the development of nanoparticle-based anti-cancer drugs and gene delivery systems denotes a favorable approach. Chemotherapeutics, currently used in the treatment of solid tumors and hematological malignancies, are systemically distributed without preferential localization to the tumor tissue, which results in high toxicity against healthily cells [2]. The employment of nanostructures as drug nanocarriers provides an effective way to minimize the side effects and to improve pharmacological properties of conventional antineoplastic agents. As the platforms for drug delivery systems liposomes, solid lipids nanoparticles, dendrimers, silicon nanostructures, polymer conjugates, micelles, carbon nanomaterials and protein or nucleic acid-based nanoparticles have been tested [30–34]. However, despite a variety of nanomaterials designed for cancer targeting, only a limited number of liposomes and polymer nanoformulations were clinically approved. Several drug-targeting strategies can be engaged to reach target tissue. Those include passive and active drug targeting and magnetic field-mediated and triggered drug delivery.

### Passive drug targeting strategy

**Fig. 2:**
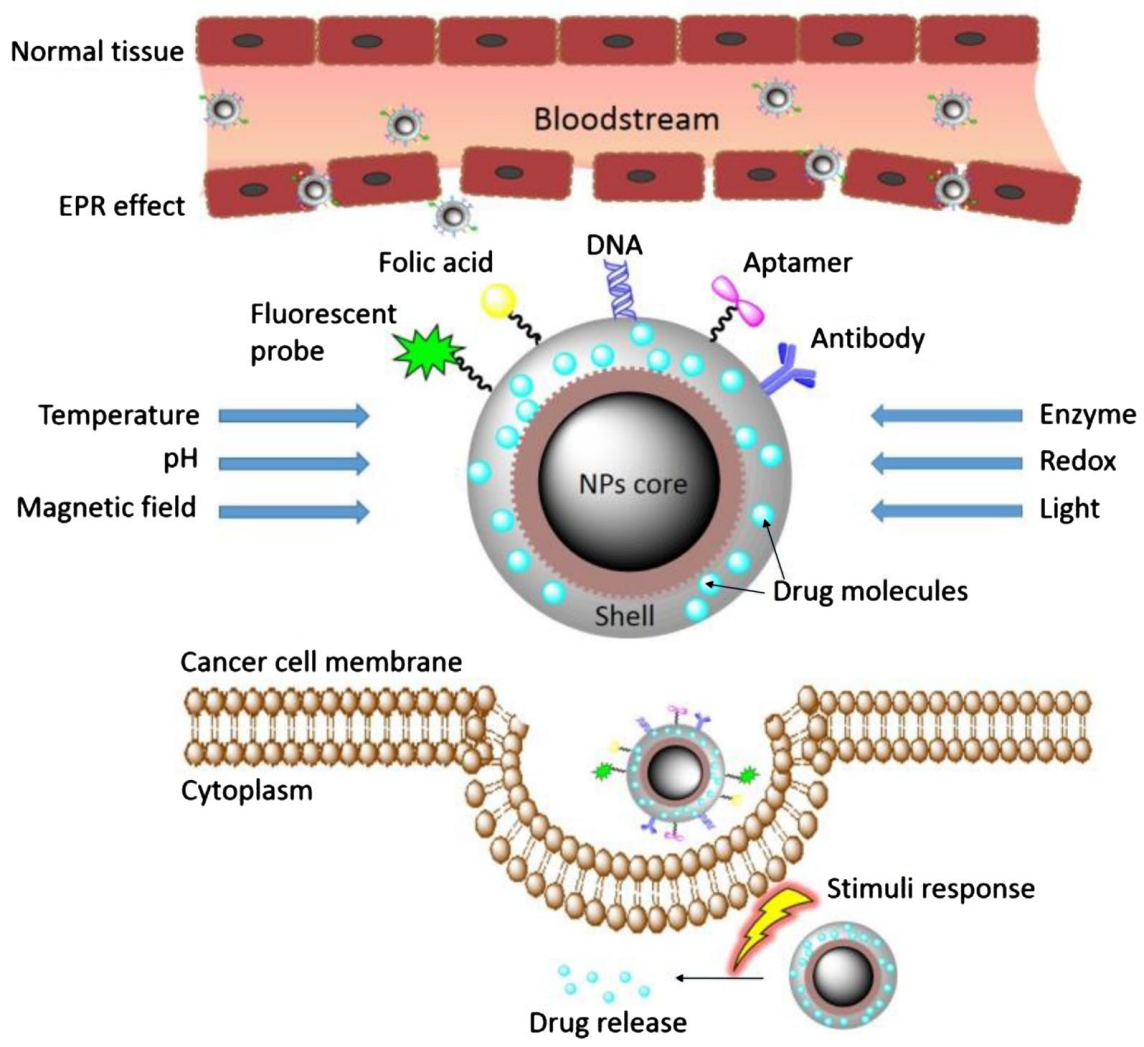
Active drug delivery using targeted ligand/moieties and stimuli-responsive nanoformulations. Figure presents the model of diblock co-polymer nanoparticles with protective cover around the core and stimuli-response shell. Passively circulated nanoparticles accumulate in tumors via enhanced permeability and retention (EPR) effect and are released into extracellular environment of tumor. The attachment of homing ligands, targeted against specific moieties on the surface of cancer cells makes available for recognition of tumor cells from normal cells. Additionally, the specificity of nanoparticles-based therapeutics might be enhanced due to employment of nanosystems sensitive to triggering by external factors, such as temperature, light, and magnetic field, alternations in pH value or as effect of biological activity of enzymes, which allows for release of factors-activated payload drugs into cancer cells via receptor-mediated endocytosis, phagocytosis, pinocytosis or macropinocytosis

The passive targeting strategy rest on preferential drug accumulation in tumor cells achieving trough enhanced vascular permeability and retention effect (EPR). The concept of this phenomena and its impact on transport of nano-drugs into cancer tissues was formulated for the first time in 1986 [35]. This theory is based on the fact, that tumor vasculature is characterized by discontinuous epithelium, impaired lymphatic drainage and reduced uptake of the interstitial fluid in contrast to normal blood vessels with firmly sealed endothelium. Subsequent accumulation of macromolecules provides the environment supporting the passive transport of nanotherapeutics to the target site [36]. High heterogeneity of EPR effect cause a significant limitation of this strategy, not only among different patients, but also in the case of the same subject, which whom a varied distribution of pore sizes and consequently, diverse drug delivery might be observed [37]. A precise impact of EPR effect on nanoparticles accumulation in tumor tissues is also difficult to determine, since a variety of nanoparticle properties, including shape, size, zeta potential, presence of homing ligands is involved in this process [38]. Previously, some drug delivery systems using this strategy were introduced to clinical trials. Such agent was SP1049C—pluronic polymeric micelle-based nanoparticles caring doxorubicin tested for advanced adenocarcinoma of the esophagus and gastroesophageal junction treatment and murine leukemia. It was reported that SP1049C decline tumorigenicity and aggressiveness of cancer in vivo and diminishes BCRP (breast cancer resistant protein) overexpression. It also modifies DNA methylation profiles, which results in sensitization of MDR cancer cells to antineoplastic treatment [39, 40]. In 2005 and 2008, SP1049C has received an orphan drug designation from FDA for the treatment of esophageal carcinoma and gastrointestinal cancer, respectively. Other nanoformulation, tested in clinical trials, was NK911—micelle encapsulated doxorubicin proposed for the treatment of various solid tumors. However, to date no development news related to SP1049C and NK911 has been reported and trials are not listed in database provided by U.S. National Institutes of Health [41]. Passive drug strategy appears to contribute as well in in vivo tumor targeting of the bile acid-conjugated chondroitin sulfate A-based nanoparticles (CSA-DOCA NPs) presented recently for the delivery of doxorubicin by Lee et al. [42]. A significant restriction in passive drug strategy is small and insufficient accumulation of drugs in target cancer tissues. It was reported, that transport of anti-cancer agents via EPR effect results in internalization into cancer cells only a small part of the injected dose [43]. In response to this limitation, active drug targeting strategy has been developed (Fig. 2).

### Active drug delivery strategy

Presence of specific homing ligands, attached on the surface of nanosystem and enabling active binding with receptors overexpressed on tumor cells represent a standard model of active drug delivery strategy. A number of molecules, including transferrin-receptors (TfR), epidermal growth factor receptors (EGFR), folate receptors (FR), CD44 or CD22 can be engaged in this manner [34, 44–47]. Accordingly, tumor-specific ligands interact with receptors on the surface of cancer tissues, triggering receptor-mediated endocytosis and internalization of nanoparticle into cancer cells [48]. Surface of nanotherapeutics can be functionalized by a number of tumor targeting agents including small molecules, peptides, monoclonal antibodies (mAb) or their fragments, aptamers and nucleic acids.

### Monoclonal antibodies

For several years the most promising class of homing ligands used in the design of targeted nanoparticles were monoclonal antibodies. They are successful in several antibodies-based anti-cancer therapeutics acting through induction of antibody-dependent cellular cytotoxicity (ADCC) and complement dependent cytotoxicity (CDC) [49]. According to studies performed by Kirpotin et al. the engagement of immunoliposomes significantly improves intracellular uptake of nano-drug, without considerable effect on tumor localization [50]. In such manner rituximab—an IgG1 antibody binding to the CD20 receptor, present on the cells of most B cell neoplasms, were tested as an example of mAb immobilized on the surface of nanomaterials [51, 52]. Studies performed by Popov et al. demonstrated that PLA-conjugated rituximab show a significant improvement in the cellular uptake and ADCCC/CDC effect when compared to non-targeted counterparts [53]. However, a number of limitations and restrictions governing the development of mAb-based nanotherapeutics, including high synthesis costs, large size, immunogenic properties, rapid nanoparticle clearance and sensitivity to environmental encounters, such as temperature, salt concentration and enzyme with subsequent poor resistance to organic solvents results in limited number of full antibody targeted nanoparticle in clinical testing [54]. In currently ongoing clinical trials antibodies’ fragments in liposome-based nanoformulations, such as C225-ILS-DOX or SGT-53, were reported. Importantly, Fab fragments make available to preserve high antigen binding specificity following by smaller size of synthetized nanoformulations and less immunogenicity [36]. Recently, Ahmed et al. demonstrated the results suggesting that immobilization of antibodies onto a surface of gold-coated nanoparticles significantly decreases the ability of cetuximab to initiate an ADCC response in EGFR-expressing H1975 tumor xenografts [55]. In contrast, cetuximab and its fragment were employed in synthesis of oxaliplatin-loaded EGFR-targeted liposomes tested for treatment of EGFR-positive colon cancer. Co-treatment with receptor-targeted antibodies and liposomes as drug delivery nanoformulations results in increased cytotoxic activity against cancer cells. Additionally, the employment of Fab’ fragments enable to eliminate the uptake by phagocytic cells resulting in better biocompatibility and in vivo efficacy [56]. Furthermore, Fan et al. provided a novel AuNPs-conjugated-rituximab-based immunodetection method to label and image specifically the CD20 on the malignant lymphoma Raji cells surface [52]. Sun et al. proved that dimercaptosuccinic acid-modified iron oxide MNPs co-loaded with anti-CD22 antibodies and doxorubicin (anti-CD22-MNPs-DOX) may be employed as drug nanocarriers in the treatment of non-Hodgkin’s lymphoma due to increased uptake of DOX and induction of apoptosis process [46].

### Aptamers

The second class of tumor targeting ligands are aptamers, defined as short single-stranded RNA or DNA sequences of oligonucleotides capable to interact with target receptors on the surface of cancer tissues with the selectivity and affinity compared with those observed for monoclonal antibodies. With respect to the unique properties of monoclonal antibodies, small size of aptamers, their decreased immunogenicity and associated with it better biodistribution and stability encourage to the development of aptamers-based nanoformulations suitable for medical application [57]. Immobilization of cancer cell-specific single-strand DNA aptamers on the nanostructures may provide an effective strategy to develop new drug delivery systems. It was demonstrated that the employment of DNA aptamers with doxorubicin-encapsulated DOTAP/DOPE nanoparticles in the treatment of breast cancer significantly suppress the tumor growth and increased the animal survival rate in xenografts model [58]. Moreover, nanoformulations presented by Roy et al. based on Fe_3_O_4_-saturated lactoferrin (Fe_3_O_4_-bLf) nanocarriers with locked nucleic acid (LNA) modified aptamers, improve the survival rate in the triple positive xenograft colon cancer model (EpCAM, CD133, CD44), due to phosphorylation of p53, induction of apoptosis and mitochondrial depolarization. In this setting a complete regression of tumor was observed in 70 % of mice. In addition to anti-cancer properties, it is hypothesized that these multi-functional nanosystems may be employed in near-infrared (NIR), MRI and CT imaging [59]. Some reports supporting the anti-cancer potential of aptamers and their utility in the active drug targeting strategy are presented in Table 1.

**Table 1:** The engagement of aptamer-based nanoformulations in anti-cancer therapy

Type	Nanoformulation	Indication	Reference
Anti-HER2 aptamer (HApt)	Gold nanoparticles	HER-2 positive breast cancer	[207]
CD133 aptamer	Salinomycin-loaded PEGylated PLGA nanoparticles	Osteosarcoma	[208]
CD133 aptamers A15 and EGFR aptamers CL4	Salinomycin-loaded PLGA nanoparticles	Hepatocellular carcinoma	[209]
EGFR-targeting aptamers	Triple-functional pRNA-3WJ nanoparticles	Triple-negative breast cancer	[210]
EpCAM aptamer	Doxorubicin-loaded PEG-PLGA nanoparticles	Non-small cell lung cancer	[211]
Mucin 1 aptamer	Gold nanoparticle-hybridized graphene oxide	Breast cancer	[212]

### Targeting ligands based on peptides, proteins and small molecules

Recently, TfR, involved in the transport of iron, necessary for cell proliferation, through the biological membrane became a biological target for transferrin-based drug nanocarriers. TfR are overexpressed in a variety of malignancies (up to 100-fold higher than the average expression in normal cells) and due to their ability to internalize into cells in a clathrin/dynamin-dependent manner and their recycling back to the cell surface, they might be employed in DDS. Importantly, targeting of TfRs can be facilitated through the use of variety drug delivery strategies, including these using transferrin, specific peptides, mAb or single chain antibody fragments specific for the extracellular domain of these receptors [60]. Transferrin-decorated PEGylated AuNPs accumulate specifically in cancer cells, in contrast to healthily liver tissues, which was investigated using Neuro2A cells-bearing mice. It was confirmed that employment of specific ligand that promote the incorporation of nanotherapeutics into neoplastic cells via receptor-mediated endocytosis results in optimized release of the drug, promoting a better therapeutic action, while limiting non-specific transport of nanocarriers to the healthy cells of the patients takes place [61]. Moreover, nanocomposite consisting of transferrin-functionalized AuNPs and graphene oxide (Tf-AuNPs/GO) was developed as NIR-based fluorescent probe for bioimaging cancer cells [62]. Eradication of breast cancer MDA-MB-231 cells was successful with transferrin-receptor engaged as target molecule for vitamin E TPGS (d-alpha-tocopheryl polyethylene glycol 1000 succinate)-based nanosystems encapsulated in the lipophilic core of the micelles [34]. Studies performed on mice brain cancer model revealed that PLGA polymeric nanocarriers might be employed for delivery of transferrin-methotrexate conjugates. Jain et al. demonstrated that development of transferrin-based nanosystems improved biocompatibility and greater anti-tumor activity against brain cancer [33]. Nanoformulation using TPGS might also be employed in treatment of doxorubicin-resistant breast cancer and in co-treatment of multidrug resistance tumors with paclitaxel and 5-fluorouracil [4, 63]. Overexpressed in a number of malignancies folate-receptors (FR) represent another promising target moiety for anti-cancer treatment. Folic acid, due to its high affinity to these receptors has been used in DDS designed for the treatment of breast cancer, as element of photodynamic therapy, when conjugated with multifunctional cobalt ferrite nanoparticles or as targeted CT contrast agents in perfluorooctylbromide nanoformulations (FR-TPNPs) for early diagnosis of ovarian cancer [25, 47, 64, 65]. The current interest of scientists focuses also on tumor-homing peptide—CREKA (Cys-Arg-Glu-Lys-Ala), recognizing fibrin-associated plasma proteins, overexpressed on cancer cell surface. Due to leaky vasculature of tumors and the presence of fibrin-fibronectin complexes within antineoplastic tissues, CREKA possess the ability to specifically target cancer cells and internalizing into their cytoplasm. The special feature of this pentapeptide includes stimulation of platelet clot formation within tumors tissues, which increase the uptake of CREKA-conjugated nanoparticles. Furthermore, nanoparticles localized in tumor cells induce additional local clotting, which results in amplified influx of further amounts of particles [66]. CREKA-based nanoformulations are currently investigated in terms of their use in the treatment of non-small cell lung cancer and breast cancer [67, 68].

An ever-growing number of studies confirmed that peptides belonging to the family of natural antimicrobial peptides (AMPs) might be employed in targeted anti-cancer therapy. It was demonstrated that LL-37 peptide proteolytically released from human cathelicidin protein (hCAP18) and its synthetic analogs (i.e. ceragenins) are characterized by a broad spectrum of pleiotropic activity, including their influence on carcinogenesis [69–72]. Previous studies established that anti-cancer potential of AMPs is determined by high membrane activity of these compounds resulting in alternation of biological membrane architecture and increased permeability. A hCAP18 based nanosystems can be used in the combination anti-cancer therapy of ovarian cancers [73]. Importantly, positively charged AMPs and its analogs possess high affinity to cancer tissues having a negative surface charge, which provides a way to distinguish neoplastic from normal cells. Our previous studies revealed, that LL-37-induced apoptosis of colon cancer DLD-1 cells can be enhanced using MNPs as drug nanocarriers [74]. The use of nanoparticles allows to design of high biocompatible ceragenins-based nanosystems, since immobilization of CSA-13 on the surface of MNPs decrease the hemolytic activity of ceragenin, observed in the case of non-conjugated compounds [75]. Data presented by Kuroda et al. indicates that these nanoformulations give great hope for effective colon cancer therapy [76, 77]. Clinically significant tumor ligands conjugated in nanoparticles systems are summarized in Table 2.

**Table 2:** Examples of target moieties in anti-cancer nanoformulations

Target moiety	Nanoformulation	Active compound	Indication	Therapy	Reference
Epidermal growth factor receptor	Peptide-targeted gold nanoparticles	Pc 4	Brain cancer	Photodynamic therapy	[213]
Epidermal growth factor receptor	PLGA nanoparticles	Tylocrebine	Several types of tumors, including epidermoid cancer	Chemotherapy	[214]
Fibrin-associated plasma proteins	CREKA-conjugated dextran-coated iron oxide nanoparticles	Iron oxide NPs	Non-small lung cancer	Hyperthermia	[67]
Fibrin-associated plasma proteins	CREKA-conjugated liposomes	Doxorubicin	Breast cancer	Chemotherapy	[68]
Folate receptors	PLGA polymeric nanoparticles	Doxorubicin	Breast cancer	Chemotherapy	[47]
Folate receptors	Cobalt ferrite nanoparticles	Hematoporphyrin	Several types of FR-positive tumors	Photodynamic therapy	[65]
Folate receptors	Deoxycholic acid-O-carboxymethylated chitosan nanoparticles	Paclitaxel	Breast cancer	Chemotherapy	[25]
IL-13Rɑ2	Liposomes	Doxorubicin	Glioblastoma multiforme	Chemotherapy	[215]
Integrin receptors	RGD-modified liposomes	Paclitaxel	Hepatocellular carcinoma	Chemotherapy	[216]
LHRH receptor	Gold nanorods	Goserelin	Prostate cancer	Radiotherapy	[217]
Transferrin receptors	PEGylated gold nanoparticles	AuNPs	Mouse neuroblastoma	Chemotherapy	[61]
Transferrin receptors	VitE TPGS-encapsulated micelles	Docetaxel	Breast cancer	Chemotherapy	[34]
Transferrin receptors	PLGA polymeric nanoparticles	Methotrexate	Brain cancer	Chemotherapy	[33]

## Employment of nanomaterials for delivery of nucleic acid-based drugs

The miRNAs play a crucial role in cancer development and might be used as therapeutic targets for novel antineoplastic agents. Recent research confirmed that miRNAs promote cancer invasion and migration, are involved in acquired chemoresistance and are considered as a predictive factor for chemotherapy response and a prognostic factor for overall survival in a variety of malignancies [78–80]. Since antisense oligonucleotides (ASOs or anti-RNAs) bind directly to miRNAs and block their biological activity, they possess the potential to be employed for anti-cancer therapy. One of ASOs is an anti-miR-150 expression vector (PR-ASO-150) constructed to inhibit proliferation of lung adenocarcinoma A549 cell line by regulating of expression of miRNA-150 [81]. In 2015 Tao et al. demonstrated that use of vector encoding ASOs against miR-21 (p-miR-21-ASO) impairs the invasion and proliferation ability of colon cancer cells. This effect was achieved due to reversed PTEN (phosphatase and tensin homolog) expression and altered the transduction of AKT and ERK pathways in cancer cells [82]. Since both of these factors is involved in tumorigenesis (PTEN is recognized as tumor suppressor and angiogenesis inhibitor and AKT/ERK pathway is associated with cancer progression) it was concluded that the employment of anti-mRNA factors might be useful for development of biological anti-cancer therapies [83]. Nevertheless, a significant limitation in ASOs-based therapy is low intratumor internalization conditioned by large molecular weight and high surface charge of ASOs, their poor stability and adverse effects induced by systemically administered anti-RNAs [84]. In order to solve these problems, a number of researchers focus on design of antisense oligonucleotides-loaded nanoparticles. Previously, it was presented that loading of human serum albumin nanoparticles with trastuzumab-modified ASOs increase their pharmacokinetic properties without affecting their impact on gene expression [85]. Costa et al. designed chlorotoxin-based nanoparticles coupled to liposomes encapsulating anti-RNAs for the modern therapy of glioma. A common mechanism of action involving the binding of chlorotoxin selectively to glioma cells (and not to health cells) and nanosystem-mediated silencing of miR-21 resulted in overexpression of PTEN and PDCD4 (programmed cell death protein 4; tumor suppressor) activation of caspase 3/7-dependent apoptosis and impaired cell proliferation [86]. Additionally, study conducted on mice model presented that nanoparticles-mediated delivery of ataxia-telangiectasia-mutated (*ATM*; radiosensitization gene) ASOs provide a way to sensitize of head and neck squamous-cell carcinoma cells to irradiation [87]. The newest studies presented by Li et al. confirmed great potential of these agents in the treatment of acute myelogenous leukemia [88]. However, a better understanding of uptake mechanism of ASOs is crucial for development of anti-RNAs as effective antineoplatic compounds. Recently, Ezzat and colleagues confirmed that mechanism of cellular uptake of some ASOs is dependent on their self-assembly into nanoparticles, forming micelles, obtaining negative charge and involves binding to class A scavenger receptor subtypes (SCARAs) [84].

The development of nanotechnology enabled as well the introduction of a number of siRNAs-based anti-cancer nanoformulations. Previously, some attempts to make the complexes of siRNAs (small interfering RNAs) with cationic lipids and polymers in order to achieve high affinity for siRNAs, proper deliver and controlled release into the cells and to protect siRNAs from degradation in in vivo conditions were persuaded. However, a significant aggregation of complexes caring positive charge in the presence of plasma proteins was followed by rapid elimination by phagocytic cells. Such effect might limit their use [89]. Presently, nanolipid-based formulations for siRNAs transport are more desirable. Interesting nanoformulation using siRNA was introduced by Shah et al. The multifunctional nanosystem based on siRNA targeted to CD44 mRNA and a synthetic analog of luteinizing hormone-releasing hormone (LHRH) peptide as a tumor-targeting moiety to transport paclitaxel conjugated on dendrimer-based nanoformulation seems very promising. Effective delivery of CD44 mRNA-targeting siRNA provides new solution to treat metastatic ovarian cancer [45].

The development of gene delivery strategies applicable in the treatment of malignancies results in design of nanocarriers for short hairpin RNA (shRNA) molecules in order to silence cancer-relevant genes. A considerable limitation in delivery of RNA interference factors is passive entry of naked shRNA into cells, unsatisfactory release of RNAi factors from carriers and escape from endosome [90]. To solve this problem, novel nanocarriers, safer than previously tested retroviral-based vectors, are needed. Lately, a number of interesting nanoparticle-based formulation for shRNA delivery have been presented. Reported combinations were tested against hepatocellular carcinoma, glioma, melanoma, ovarian and prostate cancers [91–95]. The brief summary of these reports is provided in the Table 3.

**Table 3:** Possible shRNA nanocarriers for the treatment of malignancies

Nanoformulation	Tested cancer cell lines	Results of the study	Reference
Hydroxyapatite nanoparticles-delivered plasmid-based SATB1 shRNA	Human glioma U251 cells	Significant inhibition of growth, invasion and angiogenesis, down-regulation of SATB1, cyclin D1, MMP-2 and VEGF, increased Bax and caspase-9 activity	[92]
CD44-targeted shRNA delivered by PLGA-based NPs	Human ovarian SKOV-3 cells	Inhibition of angiogenesis, proliferation of cells and the induction of apoptosis	[45]
PEI-coated gold NPs with chitosan-aconitic anhydride and shRNA	Human hepatocellular carcinoma	Enhancement of sensitivity of cancer cells to doxorubicin, induction of tumor growth, decrease of ABCG2 expression	[91]
PEG-PEI co-polymer/shRNA	Prostate cancer	Effective inhibition of EZH2 expression	[94]
jetPEI-based NPs with CXCR4 shRNA	Melanoma	Decreased expression of CXCR4 mRNA, inhibition of pulmonary metastasis of melanoma cells	[95]

*SATB1* special AT-rich sequence-binding protein-1, *PLGA* poly D, L-Lactide-co-glycolide acid, *PEI* polyethyleneimine, *PEG* polyethylene glycol, *TREM-1* triggering receptor expressed on myeloid cells-1, *EZH2* the enhancer of zeste homolog 2, *CXCR4* CXC motif chemokine receptor 4

## Triggered drug delivery by stimuli-sensitive nanoparticles

One of the active drug delivery strategies involves employment of stimuli-sensitive nanomaterials, releasing the drug in the precise target tissue due to activation by external factors or by changes in local endogenous conditions. In this strategy, during the first stage, drug is passively delivered and accumulated in tumor tissues via the EPR effect. When nanosystem reaches the target site, the nanoparticles are activated and release incorporated drugs [96]. The ever-growing number of studies confirmed that this strategy might lead to the development of new class of drug delivery systems [97]. To date, a number of stimulus factors, including light, radiofrequency (RF) energy, magnetic field, enzymes or alternation in pH value, have been explored [9, 31, 98–101]. Recently, Yingyuad et al. described new PEGylated siRNA-nanoparticles activated by human leukocyte elastase (HLE) or matrix metalloproteinase-2 (MMP-2), both present in the extracellular spaces of tumor in order to promote invasion and metastasis of cancerous cells via degradation of basement membrane and extracellular matrix barrier. The biological activity of enzymes results in cleavage of enzyme-responsive linkers and release of payload drugs to the target site. Studies performed both with breast cancer MCF-7 cells (HLE protein-positive) and primate fibroblastoma HT1080 cells (expressing MMP-2) confirmed that this formulation possess the potential for specific DDS due to controlled siRNA release. However, the exact activation mechanism is still unclear [102]. MMP-2 proteolytic activity was also used in polymer-coated mesoporous silica nanoparticles [103], in polystyrene-based nanosystems and PEGylated AuNPs conjugated with gelatin as the moiety to activate release of doxorubicin [101, 104]. Additionally, van Rijt et al. synthetized avidin-capped MSNs functionalized with linkers, exclusively cleaved by MMP9 for controlled release of cisplatin into lung tumors [105]. Lately, scientific interest has focused on the pH-activated nanosystems. A variety of pH-responding polymers, both un- and biodegradable, has been identified [106]. The employment of pH-sensitive nanocarriers is based on the cancer tissues low pH (pH ~ 6.5), especially their endosomes and lysosomes (pH 5.0–5.5) are more acidic when compared to blood physiological pH (pH ~ 7.4) [107]. Indeed, acidic conditions are required for protonation of the carboxyl group of laurate followed by decrease of the electrostatic interaction between the acid and doxorubicin, which results in release of drug from SLNs-based nanoformulations. Such were designed for treatment of DOX-resistant breast cancers. Importantly, the solubility of DOX increased in acidic environment, which improves the release rate of drug [31]. Moreover, mild acidic conditions, characteristic for tumor environment facilitate release of DOX from polymer-conjugated MSNs due to hydrolysis of the acid-sensitive acetal linkage and dissociation of polymer coating layer, protecting payload drug from release in physiological pH [108]. Recently Wei et al. presented pH-mediated release of DOX from anti-MDR-cancer nanosystems. Nanoformulation based on self-assembling amphiphilic dendrimer (AmDM) generates nanomicelles to encapsulate doxorubicin. Studies performed on DOX-resistant breast cancer MCF-7 cell line demonstrated that synthetized nanosystem exerts increased anti-proliferation effect due to rapid and effective, acidic pH-mediated cellular uptake. It was confirmed that terminal primary amines and the tertiary amines in the interior of the dendron become protonated, giving the dendrimer high positive charge and leading to improved drug release. Importantly, AmDM-based nanoparticles for effective treatment of MDR cancers required macropinocytosis process that can bypass the efflux pumps contributing to the sufficient uptake of antineoplastic agents in MDR tumors [109]. However, unspecific partial release of drugs in extracellular environment of normal cells, which results in toxic effect in place different than target cancerous tissues represents a significant limitation of this method [106]. Considering those restrictions Huang et al. designed the dual-sensitive nanosystem responding not only to alternation in pH, but also to cytoplasmic concentration of glutathione (GSH). Since it was confirmed that intracellular and extracellular tumor environments are characterized by the different concentrations of GSH (range 1–11 mM and ~10 µM, respectively), pH/GSH—co-triggered nanosystem may provide a new non-toxic, highly biocompatible system for doxorubicin delivery. In effect nanostructure was better triggered at pH 5 and 10 mM GSH concentration than in the presence of only one factor. Moreover, it was assessed that DOX internalized into HeLa cells through endocytosis process followed by drug translocation into the cells’ nuclei [110]. The appropriate concentration of GSH is also a stimulus factor for camptothecin-loaded nanoparticles [111].

Light as factor activating release of payload drugs from stimuli-responsive nanoformulations was presented in few previous studies. Nevertheless, the suitable way to accomplish drug release in physiological conditions using remote light activation and to achieve proper specificity to cancer cells is still a challenge. In 2014, Ju et al. demonstrated light-responsive nanosystem developed for controlled release of DOX using light-induced pH-jump activation and cleavage of the boronic ester linkages. This nanosystem is based on photoacid generator (PAG) producing strong acid due to illumination with UV or NIR light. Immobilization of PAG into graphene oxide-capped mesoporous silica in the presence of folic acid-modified DOX exhibited selective internalization into cancer cells without adverse effects [112]. Release of DOX from NIR light-absorbing AuNPs coating with thermally responsive poly-(N-isopropylacrylamide-co-acrylamide) hydrogel occurs in similar way [113]. Since polymeric material used in such nanosystem is characterized by a temperature-dependent alternated content of water, release of load occurs during removal of water from hydrogel surface as result of phase transition. Importantly, functionalization of AuNPs with biocompatible polymeric surface prevents aggregation of nanotherapeutics in physiological conditions, comparable to PEGylated nanoformulations. Interestingly, Oliveira et al. used doxorubicin-loaded superparaMNPs to ensure release of DOX upon application of a local high frequency magnetic field. In this system induced magnetic hyperthermia increased cytotoxicity of nanoparticles against HeLa cells [100]. Alternating magnetic field (AMF) mediated generation of heat by nanocubes provides the way to release DOX from thermo-responsive polymer-incorporated iron oxide nanocubes due to physical transition of polymer in the presence of increased local temperature [114]. This is consistent with recent study, demonstrating the release of embedded cargo as response to AMF-triggered destruction of polymer walls. Considering the limitations for in vivo application of light-induced delivery systems, particularly the strong absorption of light by tissues and restricted penetration depth of light other methods should be considered as more suitable for future clinical use. Compared to light, AMFs can penetrate deeper into cancer tissues, and it might be employed for deeper tumors treatment [115]. The generation of heat in order to release incorporated anti-cancer agents was used in radiofrequency-triggered drug release, presented as novel approach to assure targeted drug delivery. Recently, Du et al. demonstrated multi-functional nanosystem, in which conversion of radiofrequency energy into thermal energy has permitted the release of docetaxel from thermosensitive liposomes [116]. Additionally, the use of non-invasive RF-field results in disruption of pluronic F-108 in pluronic-coated ultra-short nanotubes (USNTs), which allows the release of cisplatin from nanoformulation [99]. Other stimuli-sensitive nanoformulations demonstrated in 2015 are summarized in the Table 4.

**Table 4:** Examples of stimuli-responsive nanotherapeutics

Stimulus factor	Nanoformulation	Active compound	Tested cancer cell lines	Reference
AMF	Iron oxide/gold nanoparticles	DNA	Human cervical HeLa cells	[218]
GSH	PEGylated, RGD-modified, and DSPEIs-functionalized gold nanorods	shRNA	Human glioblastoma U-87 MG-GFP cells	[219]
GSH	mPEGylated PLA-conjugated micelles	Curcumin	Human cervical HeLa cells	[220]
Light	Bridged silsesquioxane nanoparticles	Plasmid DNA	Human cervical HeLa cells	[221]
Light	Micelles	Cisplatin prodrug and cyanine dye (Cypate)	Cisplatin-resistant lung cancer A549 cells	[222]
Light/pH	Chitosan derivative-coated CNTs encapsulated in nanogel	Doxorubicin	Human cervical HeLa cells	[223]
pH	mPEGylated PLGA-P-Glutamic acid nanoparticles	Doxorubicin	Lung cancer NCI-H460 cells, breast cancer MCF-7 cells	[224]
pH	Multifunctional amphiphilic block copolymer containing cyclic orthoester and galactose groups	Doxorubicin	Liver hepatocellular carcinoma HepG2 cells	[225]
pH	Porous bowl-like PLA-modified MSNs	Doxorubicin	Gastric cancer HGC-27 cells	[226]
pH/GSH	Multi-layered nanocomplexes	Doxorubicin, siRNA	Human hepatocarcinoma QGY-7703 cells	[227]

Multifunctional “smart” nanoparticles carrying drugs targeted preferentially to the cancer cells will lead to development of better treatment for patients with cancer

## Nanoparticles-based approaches to reverse multidrug resistance of cancer cells

The numerous mechanisms were reported as involved in induction of multidrug resistance (MDR) in different cancer cells. Overexpression of ATP-dependent efflux pumps, mainly glycoprotein P (P-gp), breast cancer resistant protein (BCRP), multidrug resistance associated protein (MRP), decreased drug uptake via activation of surface transporters, alternations in apoptotic pathway, increased capacity to drug-induced DNA repair and activation of detoxification systems resulting in augmented drug elimination should be consider as a mechanism leading to MDR [117]. Among them, efflux pumps-mediated resistance is most clinically significant, given into account the number of studies reporting drug-induced overexpression of MDR proteins as result of chemotherapy-based treatment and emerging of inflammation in the cancerous tissues [118, 119]. Importantly, over 50 % of presently used cytotoxic drugs are transported through cell membrane using these proteins, which significantly hampers the conduction of effective systemic chemotherapy [120–123]. It is hypothesized that nanotechnology provides the way to overcome ATP-proteins-induced drug resistance due to employment of nanoparticles as drug delivery systems, ensuring sufficiently high concentration of drug in intracellular environment that permit to omit the cancer cell resistance [124]. The potential of nanoparticles as modern nanocarriers have been presented in previous sections (see: Targeted drug delivery using nanomaterials as the method to overcome lack of selectivity of conventional chemotherapeutics). Novel nanotechnology-based approaches toward treatment of MDR cancers assume the engagement of nanoparticles in order to increase intracellular drug accumulation, silence of efflux transporters genes and inhibit MDR-associated proteins and factors.

### Enhancement of intracellular drug retention

One of the most benefited method for reducing tumors resistance was developed with increased of the intracellular concentration of anti-cancer drugs that improved their therapeutic efficiency. A doxorubicin-containing nanoparticles synthetized using folate-terminated polyrotaxanes (as a drug carrier) and dequalinium (as a compound for selective delivery of drug into mitochondria) were recently described [125]. This nanosystem shows the potential for treatment of doxorubicin-resistant MCF-7 breast cancer cells and MCF-7/Adr xenografts in nude mice due to caspase-dependent mechanisms involving the activation of Bax (Bcl-2-associated X protein) and Bid (Bax-like BH3 protein) and inhibition of Bcl-2 protein. The sixfold increase of DOX intracellular uptake and decreased drug efflux for functional nanocarriers when compare to the free compounds, was reported [125]. Mitochondrial-targeting liposomes were also efficient in the therapy of paclitaxel-resistance lung cancer (A549/Taxol cell line) as demonstrated by Jiang et al. Encapsulation of paclitaxel into pH-triggered nanosystem resulted in caspase-dependent apoptosis of drug-resistant cancer cells and inhibition of growth of xenografted lung tumors due to facilitated cellular uptake and increased drug accumulation [126]. Enhanced effective drug concentration enables also the drug resistance in DOX-resistant breast cancer cells without the increased systemic toxicity [127]. Additionally, it was confirmed that nanoparticles-mediated delivery of paclitaxel into resistant P-gp-overexpressing cancer cells (KB-8-5) minimize its resistant phenotype, which is accomplished with enhanced cellular accumulation and retention of drug. Prolonged retention within tumor tissues was also confirmed in xenografted cancer model, which was linked with higher degree of microtubule stabilization, mitotic arrest, antiangiogenic activity, and inhibition of cell proliferation [128].

### Silencing of drug resistance genes

The employment of siRNAs leading to cleavage and degradation of target mRNA is a very promising approach in anti-cancer therapy. *MDR1* encoding P-glycoprotein (P-gp), is one of the major targeted gene in the treatment of drug-resistance cancers [129]. It is generally established, that silencing of *MDR1* gene followed by decrease biosynthesis of P-gp results in increased tumors sensitivity [130]. Proper delivery of siRNAs by lipid-based nanoparticles or encapsulation of siRNA into PEGylated nanoliposomes, both lead to down-regulation of P-gp expression in doxorubicin-resistant breast cancer MCF-7 cells [89]. Accordingly, employment of siRNA-based nanoformulation possess the potential to treat resistant tumors [131]. Interestingly, empty nanocarriers significantly increase the expression of P-gp.

*Mac2* gene encoding mitotic arrest deficiency protein 2 (Mac2) represents another target gene for siRNA-based interventions. Mac2 expression correlates with resistance of cancer cells to a variety of antineoplastic agents, including paclitaxel and cisplatin [132, 133]. Interestingly, complete silence of gene lead to chromosome missegregation-modulated cells death [134]. Considering these reports Nascimento et al. used EGFR-targeted chitosan nanoparticles to transport *Mac2*-aimed siRNA. They demonstrated that siRNA-based nanoformulation efficiently knock-down *Mac2* gene causing apoptosis of non-small cells lung cancer cells, which is additionally improved by controlled delivery of siRNA using EGFR-targeted homing ligand [135].

### Inhibition of MDR-associated proteins and efflux pumps

Decreasing activity of MDR-proteins and transporters responsible for efflux of cytotoxic drugs from cancer cells represents a major goal on the ways to deal with multidrug resistance in malignant tumors. It was proved, that the employment of Poloxamer 235 as inhibitor of P-gp pump results in increased permeability of various P-gp dependent drugs in resistant tumors due to its strong pore-forming properties [136]. Conducted studies confirmed that Poloxamers possess the ability to sensitize MDR cancer cells to various anti-cancer agents via incorporation into biological membrane and subsequent influence on intracellular functions, including mitochondrial respiration and synthesis of ATP, which is essential for the proper activity of ABC-dependent efflux transport proteins [137]. Tang and co-workers demonstrated that docetaxel-loaded PLGA–TPGS/Poloxamer 235 nanoparticles possess great potential to be used as novel agent to treat drug-resistant breast cancer, since its use results in greater incorporation of docetaxel in MDR cancer cells, than both docetaxel and PLGA-TPGS nanoparticles alone [4]. It is also effective to use well-characterized first and third generation of P-gp inhibitors, verapamil and elacridar, to decrease the activity of efflux transporters. Singh et al. reported that treatment with nanoformulation containing doxorubicin-loaded cationic surfactant-based nanoparticles with P-gp inhibitor encapsulated within it results in sensitization of DOX-resistant ovarian cancer cells, which was determined by interaction of positively charged nanoparticles with negatively charged cell cancer surface and simultaneous caveolae-dependent endocytosis of nanosystem [138]. Moreover, Xu et al. demonstrated promising results obtained during treatment of drug-resistant A549 lung cancer cell with PLGA-based nanoparticles loaded with cyclosporin A and encapsulated P-gp inhibitor [139]. These observations are in great agreement with reports demonstrated in previous years [140, 141].

## The employment of nanoformulations to modulate pharmacokinetic properties of cytotoxic drugs

A number of studies report that nanotechnology provide an effective way to improve pharmacokinetic parameters of cytotoxic therapeutics employed in the systemic chemotherapy of malignancies. Application of nanotechnology provides the opportunity to influence cytotoxic drug low solubility in water, hydrophobic properties, the short half-life and rapid clearance. Oil in water (O/W) nanoemulsions, offer the improvement of chemical, enzymatic and colloidal stability of carried hydrophobic therapeutics [142]. In 2014 core-matched nanoemulsions (NEs) designed to co-deliver drugs of various chemical nature was demonstrated. Functionalization of NE with vitamin E and TPGS facilitated delivery of both hydrophobic (paclitaxel) and hydrophilic (5-fluorouracil) drugs and synergism to overcome paclitaxel resistance in MDR human epidermal carcinoma cell line KB-8-5 and mouse xenograft model [63, 142]. Conducted studies confirmed that drugs encapsulation into nanoemulsion results in good stability, longer drugs circulation in blood, lower clearance and metabolism followed by higher accumulation in cancer tissues [63]. Deol et al. engaged FDA-approved, low toxic and non-immunogenic dendrimers to design water-soluble dendron–conjugated gold nanoparticles (Den-AuNPs) in order to improve stability of AuNPs in water solutions [143]. Despite having the significant anti-cancer potential, the medical use of gold-based nanotherapeutics is expressively reduced due to poor biocompatibility of these structures determined by low colloidal stability required for the prevention of AuNPs agglomeration under physiological conditions [144]. As expected, the functionalization of gold-coated nanoparticles with dendrons prevent aggregation in biological fluids in broad spectrum of pH, in contrast to non-functionalized AuNPs irreversibly aggregated in acidic pH. Additionally, the presence of large dendron ligands protect nanoparticles core from the ionic disruptions resulting in their good stability and solubility in the aqueous solution with the salt concentration of up to 100 mM [143]. There is also urgent need to improve biodistribution of paclitaxel, whose great antineoplastic properties are limited due to its insolubility in aqueous media and acquired cells chemoresistance. To solve this issue, paclitaxel was formulated at high concentration in Cremophor EL (Taxol^®^). However, with respect to its special features necessary to solubilize paclitaxel, cremophor EL-based formulation cause a variety of adverse side effects, including allergic reactions, nephrotoxicity and precipitation in aqueous solution [145]. In order to achieve sufficient drug water-solubility, without concurrent side effects, several nanotechnology-based strategies, including micellization, employment of liposomes and non-liposomal nanoparticles have been investigated [25, 26, 146]. Innovative approach to improve water-solubility of paclitaxel lead to synthesis of nanoparticle conjugates formulated by covalent attachment of drug to gold nanoparticles via DNA linkers presented in 2011 by Zhang et al. The solubility of paclitaxel in aqueous buffer increased over 50-fold when compared to unconjugated drug. Additionally, fluorophore-based labeling of DNA linkers allows for visualization of conjugates within the cells [147].

**Fig. 3:**
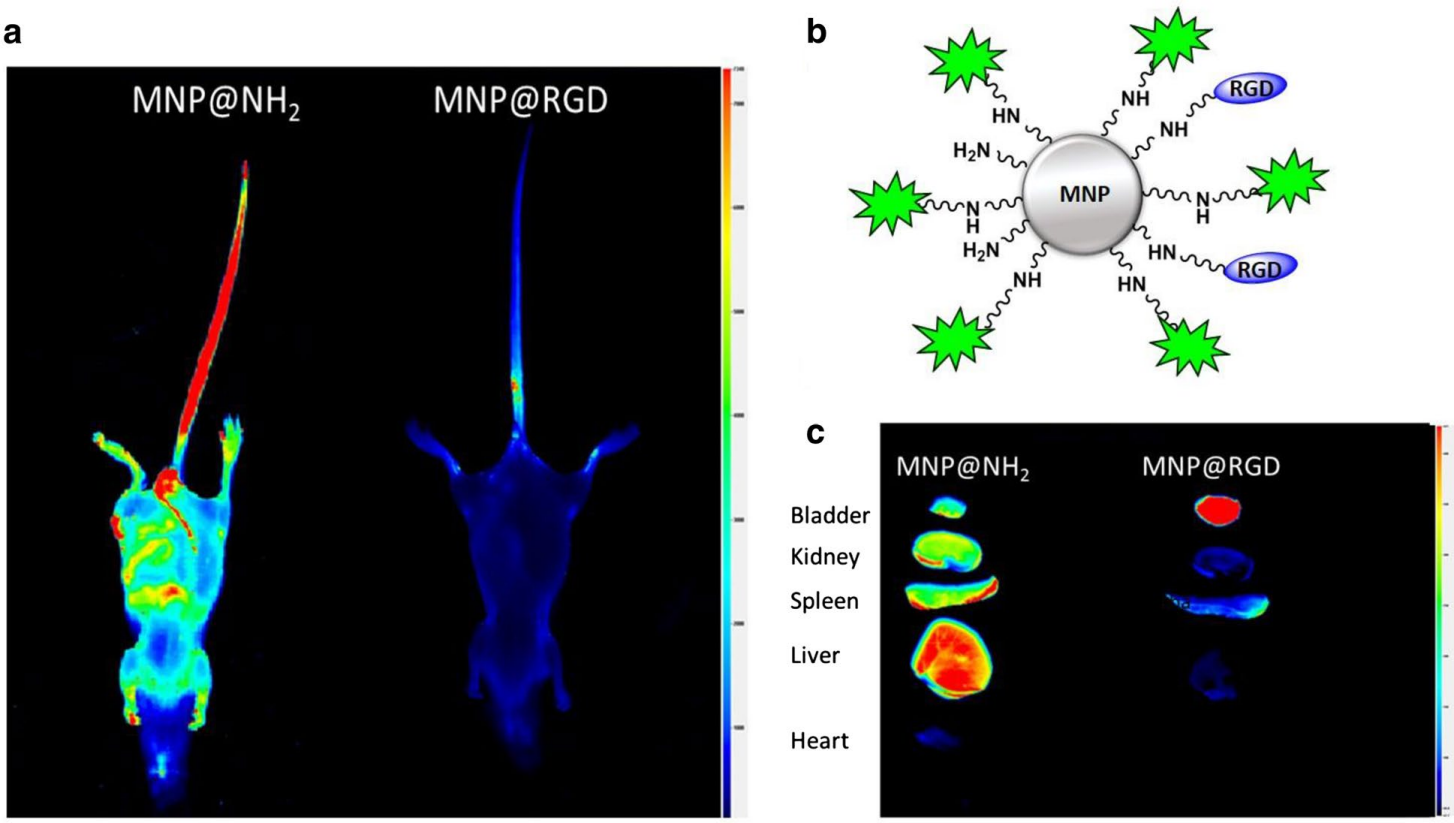
Magnetic nanoparticles (MNPs) functionalization by homing molecules (RGD-peptide) increases particles elimination and prevents non-specific accumulation in mice healthy organs. Pharmacokinetic of aminosilane coated magnetic nanoparticles (MNP@NH2) and their derivatives functionalized by RGD peptide (MNP@RGD) 8 h after intravenous injection (**a**). Structure of magnetic nanoparticles functionalized by RGD peptide and fluorescent probe DYE 800 CW (**b**). Biodistribution of MNP@NH_2_ and MNP@RGD 8 h after intravenous injection (**c**)

Rapid development of nanotechnology provides possibility to improve biodistribution and in vivo effectiveness of DNA-conjugated nanoparticles designed as controlled gene delivery systems characterized by low toxicity, good biocompatibility, biological activity and ability to enter cancer cells via scavenger receptor-mediated endocytosis [148]. Nevertheless, significant limitation using these systems in anti-cancer therapy is non-specific interaction of DNA-conjugated nanostructures with plasma proteins, resulting in nuclease-mediated rapid clearance [149]. Recently, it was reported that terminal PEGylation of the complementary DNA strand in DNA/AuNPs conjugates offers the approach to overcome adsorption to serum proteins and determine the resistance of nanosystem against DNase I-based enzymatic digestion without the affecting of cellular uptake of loaded drugs [150]. Apart from this reports, Yang et al. proved that engagement of isolated exosomes, a class of membrane secreted lipid vesicles that carry proteins and RNA among the cells, possess the great potential to delivery drugs across the blood–brain barrier and into brain via receptor-mediated endocytosis [151, 152]. To date, exosomes isolated from cell lines were employed mainly as nanocarriers to transport siRNA into cancerous cells and to deliver curcumin into target tumor tissues [153]. Studies performed on human glioblastoma-astrocytoma U-87 MG cells confirmed that loading of exosomes with doxorubicin and paclitaxel allows the drugs transport through blood–brain barrier, in contrast to non-functionalized drugs, that do not show brain uptake at all. Exosome-induced brain delivery was confirmed using zebrafish (*Danio rerio*) xenograft model. Still, there is urgent need for further studies that will evaluate the biocompatibility and effectiveness of these novel nanotherapeutics [152]. Additionally, in vivo studies performed by our research team confirmed that functionalization of RGD peptide (involved with the binding of proteins to cell surfaces) on the surface of MNPs considerably alters kinetic parameters of peptide and changes the way of drug excretion from the body of the mouse (Fig. 3).

## Physical approaches based on nanoparticles as adjuvants in anti-cancer therapy

### Magnetic fluid hyperthermia (MFH)

Theranostic potential of nanomaterials assure employment of nanostructures in a wide range of medical application, including drug delivery systems, disease imaging and therapy. A number of studies confirmed that a defined subgroup of nanoparticles, mainly superparamagnetic nanoparticles (MNPs), due to their ability to release heat in the presence of alternating magnetic field might be used in cancer therapy. It was demonstrated, that MNP-induced hyperthermic conditions (40–43 °C) leading to enhanced cancer cell death in the process of so-called MFH sensitize tumors to radio- and chemotherapy and might serve as adjuvant anti-cancer agent or as multifunctional nanosystems component to administer together with other cancer treatment modalities [154]. Magnetic nanoparticles have been tested in clinical trials as thermoablation method treatment of prostate cancer [155]. Numerous factors are involved in the process of hyperthermia-mediated cell death and the mechanism of MFH-induced killing of tumors is still under investigation. According to the latest studies changes in cell membrane fluidity resulting in greater permeability and susceptibility to antineoplastic agents, thermal degradation of BRCA2 (breast cancer susceptibility protein 2), involved in repair of damaged DNA and increase in the amount of aggregated proteins and microtubule disruption-mediated proteotoxic stress play a major role in successful eradication of cancer cell using this method [156–159]. Previously, it was reported that MFH-induced decrease in the viability of melanoma and non-melanoma skin cancer cells results from necrosis and endoplasmic reticulum-mediated apoptosis, since activation of caspase 3/7, but no caspase 8 or 9 has been observed [160]. Current hyperthermia treatments are limited when tumors are described as deep-seated and by the risk of damage of health organ surrounding tumor tissues. In contrast, an oxygen-independent mechanism of MFH-mediated killing is based mainly on protein degradation and dysfunction of cells membranes [161, 162].

Recently, Kossatz et al. synthetized magnetic nanoparticles multifunctionalized with N6L, Nucant multivalent pseudopeptide targeting a nucleolin-receptor complex overexpressed at the cancer cell surface, and DOX for combined therapy linking MFH and anti-cancer drug delivery. Use of tumor-specific N6L and DOX on the MNPs surface to increase intracellular uptake help to achieve both hyperthermia- and DOX-induced cytotoxicity [163]. The combined therapy using hyperthermia induced by gold nanorods and cisplatin-mediated chemotherapy against SKOV3 ovarian cancer cells gave also the promising results [164]. Synergistic effect between MFH and cisplatin-induced apoptosis against non-small cell lung cancer cells was described as well. CREKA-based nanoplatform to deliver cisplatin into cancer cells was designed. It was shown that CREKA-conjugated iron oxide nanoparticles increased the cisplatin-mediated cytotoxicity due to hyperthermic conditions [67].

### Photodynamic (PDT) and phototermal therapy (PTT)

Improvement of PDT selectivity and efficiency, due to employment of magnetic nanoparticles and other nanomaterials, was for a long time a main goal for scientists engaged in anti-cancer therapy. Photodynamic therapy, based on the selective photosensitizer internalization into cancer cells in order to eliminate tumor tissue using specific wavelength irradiation seems one of the most interesting anti-cancer strategies in recent years. Photosensiters-induced cell death occurs as the result of apoptosis, autophagy and necrosis process. However different factors are involved in PDT-mediated cancer cells killing. The list includes reactive oxygen species (ROS) and singlet oxygen (^1^O_2_) generation, Bax activation and caspase-dependent internucleosomal DNA cleavage followed by tumor-vasculature damage and activation of anti-cancer immune response [165]. In effect, nanoparticles acting as energy converters, transporters, and selective nanocarriers for photosensitizers, possess the great potential for use in synergistic antineoplastic therapy or as independent PDT components against skin, head and neck, cervical, bladder, prostate, brain and lung cancers [166–172].

Photothermal therapy represents an extension of PDT, where NIR-light is used to induce heat and rise of local temperature within tumor tissues leading to photoablation of the cells and cell death in oxygen-independent mechanism. Importantly, PTT allows the use of longer wavelength light than PDT (650–900 nm), which is less damaging for normal tissues [173]. Among a number of nanomaterials tested for their use in photothermal therapy, gold nanoparticles (AuNPs) took a special place. Comprehensive research on gold-based nanomaterials properties proved that flower-like and core–shell AuNPs due to localized surface plasmon resonance (LSPR) are great candidates for the employment in PTT [174]. According to the latest studies, targeted photothermal therapy using gold nanoparticles conjugated with anti-Mucin 7 antibodies might be useful in adjuvant therapy of urothelial cancer [175]. The recruitment of antibodies directed against mucin 7, being a urinary marker in bladder cancers, confirming the specificity of nanosystem and decrease the damage of health tissues, when compared to simple heating procedures used in hyperthermia [176]. Promising results were reported by Trinidad et al. They present gold nanoparticles as the multifunctional system allowing the combined photodynamic and photothermal therapy against head and neck cancer. Enhancement of efficacy of such treatment was obtained through employment of macrophages, possessing affinity to hypoxic and necrotic cells within neoplastic tissues, survived after PTT [177]. Another synergistic therapeutic option, using simultaneously PDT and PTT, was demonstrated by Fan et al. in 2014. They used nanoplatform built from A9 RNA aptamer modified with methylene blue attached to PEGylated iron core within gold shell nanoparticles. Specificity of A9 aptamer to prostate specific membrane antigen (PSMA) make possible to distinguish prostate cancer cells from normal tissues. Conducted studies confirmed that formation of ^1^O_2_ by methylene blue followed by heat generation by gold nanoparticles results in significant decrease of *LNCap* cancer viability [178]. Reports demonstrated by other authors established that combined PDT/PTT therapy using various nanomaterials, including photostable micelles gives great hope for effective antineoplastic therapy [179].

### Radiosensitization by high-Z nanoparticles

Although the radiation is highly non-selective therapeutic method, it has become one of the most important therapeutic alternatives for patients diagnosed with malignancies. In analogy to the chemotherapy, one of the greatest challenges is to deliver a lethal dose of radiation to tumor environment within tolerance of healthy tissues. It was confirmed, that nanoparticles composed of high atomic (Z) numbers due to direct interaction with ionizing radiation act as radiosensitizers and allow for the heightening of therapeutic efficacy without increasing damages to the nearby healthy tissues. It is proposed, that mechanism of such interaction involves the enhancement of the photoelectric and Compton effects followed by the subsequent emissions of secondary electrons [180]. The most studies nanomaterials, whose properties make available to intensify production of secondary electrons and ROS and in that manner enhance radiation therapy effects are AuNPs, lanthanide-based NPs, titanium oxide nanotubes and cadmium selenide quantum dots [181–183]. Noteworthy, the unique properties of nanoparticles allows the use of additional advantages of nanomaterials without sacrificing their radiosensitizing properties. Le Duc et al. proposed the employment as radiosensitizers gadolinium-based nanoparticles, since they possess high Z number and possess the potential to be used as contrast agents in MRI [184]. The additional advantage of the employment of AgNPs might be enhanced Raman scattering and antimicrobial properties [185].

A significant disadvantage of using nanoparticles as components of radiation therapy is its high sensitivity to a number of physicochemical and pharmacological parameters, such as irradiation energy, nanoparticle size, their concentration and localization in tumor tissues. Oxygen concentration present in the tumor environment represents a significant limitation in the employment of nanoparticles in radiation therapy. In 2014, it was demonstrated that radiosensitizing properties of gold nanoparticles are correlated with the level of oxygen and is lower under hypoxic than oxic conditions [186].

## Nanoformulations in recent clinical trials

Unique properties of nanomaterials makes available to employ them as effective antineoplastic agents or as a compound of combined therapy, in order to improve therapeutic effectiveness of existing anti-cancer drugs. However, despite considerable amounts of described nanotechnology-based formulations, only a limited number of them was introduced into clinical trials. Recently, the interest of the researchers has focused on the employment of already used, FDA-approved nanodrugs (Abraxane^®^, Doxil^®^, Genexol-PM^®^) as the adjuvants in combinatory therapy of malignancies. To date, Abraxane^®^, e.g. paclitaxel albumin-stabilized nanoparticle formulation (*nab*-paclitaxel) was approved for treatment of metastatic breast cancer [187]. There is more than 160 ongoing clinical trials involving *nab*-paclitaxel in combinatory therapy with other anti-cancer agents and focused primarily on the treatment of breast, lung or digestive and endocrine system neoplasms (according to the data provided by U.S. National Institutes of Health) [155]. Although some of them were terminated due to lack of funding, frequent dose adjustments or difficulties in determination of optimal dose, it was presented as well that *nab*–paclitaxel with other antineoplastic agents might provide the way to overcome drug resistance of malignancies [188, 189]. Results of phase I study described by Tsurutani et al. revealed that *nab*-paclitaxel in combination with S-1 (tegafur + 5-chloro-2.4-dihydrooxypyridine + oteracil potassium) may be promising therapy for patients diagnosed with HER2-negative breast cancer [190]. In opposition, combined treatment with *nab*-paclitaxel with tigatuzumab does not result in higher objective response rate and progression-free survival [191]. Similarly, liposomal doxorubicin (Doxil^®^), registered for treatment of HIV-related Kaposi sarcoma, metastatic breast and ovarian cancer, is tested currently against recurrent, platinum-sensitive ovarian, primary peritoneal, and fallopian tube cancer when combined with carboplatin, bevacizumab and veliparib and for newly-diagnosed multiple myeloma in combination with bortezomib [192, 193]. Results of clinical trials demonstrated recently are briefly summarized in Table 5.

**Table 5:** Results of nanoformulations in clinical—examples of nanoformulations tested in recent clinical trials

Nanoformulation	Phase of development	Indication	Conclusions from clinical trials	Reference
Liposome-encapsulated irinotecan (PEP02, MM-398)	Phase I	Advanced solid tumors	Improved pharmacokinetics and tumor bio-distribution of the free drug	[228]
Liposome-encapsulated irinotecan (NAPOLI-1)	Phase III	Gemcitabine-refractory metastatic pancreatic cancer	Extended survival with a controllable safety profile in combination with fluorouracil and folinic acid	[229]
*Nab*-paclitaxel with carboplatin	Phase II^a^	Extensive-stage small cell lung cancer	Activity against ES-SCLC, but patients required frequent dose adjustments and treatment delays	[188]
*Nab*-paclitaxel with sirolimus	Phase Ib^a^	Advanced solid tumors	Acceptable safety profile of sirolimus with nab-paclitaxel	[230]
Nanoparticulate paclitaxel	Phase I	Peritoneal malignancies	Low peritoneal clearance, minimal toxicity of treatment	[198]
Genexol-PM^®^	Phase II	Non-small cell lung cancer	Anti-tumor activity when combined with gemcitabine, but frequent 3/4 grade hematological toxicity was observed	[199]
Paclitaxel bound to poly-l-glutamic acid (Paclitaxel poliglumex) with capecitabine	Phase II	Metastatic breast cancer	Tolerable and effective treatment, but the combination failed to reach efficacy endpoint	[231]
Paclitaxel bound to poly-l-glutamic acid (Paclitaxel poliglumex) with radiation therapy	Phase II	Glioblastoma Without MGMT Methylation	Progression-free survival and overall survival was not improved	[232]
Doxil^®^ with carboplatin, bevacizumab and veliparib	Phase I	Platinum-sensitive ovarian, primary peritoneal, and fallopian tube cancer	Lower doses of veliparib will need to be considered when given in combination with platinum-based therapies, dose limiting toxicity was noted	[192]
Doxil^®^/carboplatin with tocilizumab	Phase I	Recurrent epithelial ovarian cancer	Acceptable safety profile	[194]
PLD plus cyclophosphamide followed by paclitaxel	Phase II	Breast cancer	Effective and safe treatment for patients prone to conventional doxorubicin-induced cardiotoxicity	[197]
Myocet^®^	Phase I	Glioma	The maximum recommended dose was determined. Safety profile will be studied in further trials	[200]
PLD plus irinotecan	Phase I	Ovarian cancer	High tolerance to treatment	[195]

*PLD* pegylated liposomal doxorubicin

^a^ Study terminated

It is noteworthy that results obtained from toxicity studies are highly variable. Recently, acceptable safety profile was demonstrated for combined treatment of recurrent epithelial ovarian cancer with carboplatin/liposomal doxorubicin with tocilizumab (i.e. an anti-IL-6R monoclonal antibody) and for combination of liposomal doxorubicin with irinotecan [194, 195]. The highly active combination of cyclophosphamide, bortezomib, pegylated liposomal doxorubicin, and dexamethasone (CVDD) was also well tolerated by patients with multiple myeloma [196]. Notably, the enrollment of pegylated liposomal doxorubicin with cyclophosphamide followed by paclitaxel was safe even for patients prone to cardiotoxicity as presented in results of phase II CAPRICE study [197]. Moreover, treatment of peritoneal malignancies with nanoparticulate paclitaxel have not induced toxic effects with low peritoneal clearance of drug preserved at the same time [198]. In contrast to these reports, the investigation of safety of Genexol-PM^®^ i.e. Cremorphor EL-free polymeric micelle nanoformulation of paclitaxel combined with gemcitabine in patients with non-small cell lung cancer revealed frequent hematological toxic effects [199]. The safety profile of Myocet^®^ (non-pegylated liposomal doxorubicin approved for treatment EGFR2-positive metastatic breast cancer) in children with high-grade glioma need to be studied as well [200].

## Limitations facing nanoparticles-based anti-cancer therapies

It is undeniable that nanotechnology provides a variety of novel therapeutic options applicable in the treatment of solid tumor and hematological malignancies. However, this enthusiasm must be suppressed due to numerous reports on the considerable limitations facing nanotechnology-based anti-cancer therapies. First of all, physicochemical properties of tested nanomaterials (i.e. its size, surface properties, zeta potential) influences greatly the stability in physiological fluids, their polydispersity, binding to blood proteins and associated efficiency of designed nanoformulation [14, 15]. However, the tumor accumulation and pharmacokinetics properties are not so easily to predict, even when the same polymers and elements of nanosystem are used. Similarly, they are no strong tendencies, when nanoformulations consist of particles with resembling size and shape are compared [201]. Overall, there is urgent need to recognize the exact properties of nanoparticles, which permit for maximum uptake and accumulation of drug in the target tissues.

Importantly, the unique properties of nanomaterials do not only condition their employment in therapy of cancers, but are also responsible for a variety of toxic effects. Despite the fact, that immobilization of anti-cancer agents on the surface of nanomaterials should improve their biocompatibility, it is confirmed that some nanoparticles can cause toxic effects in healthy cells [75]. It was presented that SPIONs are potentially involved in cellular toxicity (generation of ROS, impairment of mitochondrial function, inflammation and formation of apoptotic bodies), alternations in gene expression and iron homeostasis and disturbances of cell cycle regulation [202]. In 2005, it was reported that intratracheal administration of multi-wall carbon nanotubes resulted in dose-dependent increase in inflammatory factors and fibrotic reactions, followed by the accumulation of CNT agglomerates in the airways [203]. Systemic immunosuppression after 14-day exposure to inhaled multi-wall carbon nanotubes was reported by Mitchell et al. [204]. This confirms that comprehensive and detailed analysis of physicochemical properties and safety profiles of nanomaterials must be performed before their introduction to further studies and clinical applications [204].

Apart from this, heterogeneity of cancer tumors and the development of MDR phenotypes represent a final challenge to effective antineoplastic strategy, since the cancer cells within tumor differ from their xenografts counterparts. Importantly, many physiological barriers, both extra- and intracellular reduces the total quantity of nanoparticles accumulated in the tumor. Additionally, despite the great potential of nanomaterials to treat solid tumors, the treatment of metastatic cancers is still challenging, since metastasized cells are too small to create microenvironment and EPR effect is less important [205].

It is established as well, that lack of standardization of preclinical research is recognized as considerable limitation in design formulations applicable in the clinical settings. A comprehensive analysis of available data confirmed that a number of factors, including the choice of xenograft cell line, range of used experimental controls, inconsistent pharmacokinetic data, size of tested nanosystem and its dose, variation in tumor characteristics e.g. size and vascularization influences the proper assessment of delivery systems’ efficiency [201]. In order to solve this problem it is proposed that pre-clinical trials should be performed on standardized xenograft model, since differences in physiology results in alternations in circulation and drug accumulation, and tumor biology [206]. Dawidczyk et al. recommended standardization of pre-clinical studies, including the introduction of standard cell line in xenografts model, reporting tumor accumulation as % ID (the percentage of administrated nanoparticles) and performing the studies using tumor with specific type, size and unification of detection methods [201].

## Conclusions

The rapid advance in nanotechnology provides different tools to develop new anti-cancer strategies. Employment of nanotechnology-based therapeutics should in near future overcome limitation of cancer surgery, radiation and chemotherapy. Nanomaterials possess the potential to be used as effective and selective antineoplastic agents for multidrug resistant cancers. Although many of designed nanoformulations have not led to clinical success after their introduction into clinical trials, several of them provide hope for new therapeutic option in the treatment of malignancies. Current methods for the synthesis and analysis of nanosystems’ physicochemical and biological properties allows for comprehensive examination of their mechanism of action, as well as their effect on the living organism. Nevertheless, a significant diversity of nanomaterials, their specific physicochemical properties and a variety of effects on cellular processes, results in unexpected interactions and toxicity in in vivo settings. Despite this, it is expected that growing interest in nanotechnology-based anti-cancer approaches will result in solutions that will soon be used as a part of modern, efficient and individualized antineoplastic therapy.
